# A Lifetime’s Adventure in Extracellular K^+^ Regulation: The Scottish Connection

**DOI:** 10.1007/s11064-017-2319-4

**Published:** 2017-06-21

**Authors:** Angus M. Brown

**Affiliations:** 1 School of Life Sciences, University of Nottingham, Nottingham, NG7 2UH UK; 2 Department of Neurology, University of Washington, Seattle, WA 98195 USA

**Keywords:** Potassium, Astrocyte, Nernst equation, Buffering

## Abstract

In a career that has spanned 45 years and shows no signs of slowing down, Dr Bruce Ransom has devoted considerable time and energy to studying regulation of interstitial K^+^. When Bruce commenced his studies in 1969 virtually nothing was known of the functions of glial cells, but Bruce’s research contributed to the physiological assignation of function to mammalian astrocytes, namely interstitial K^+^ buffering. The experiments that I describe in this review concern the response of the membrane potential (Em) of in vivo cat cortical astrocytes to changes in [K^+^]_o_, an experimental manoeuvre that was achieved in two different ways. The first involved recording the Em of an astrocyte while the initial aCSF was switched to one with different K^+^, whereas in the second series of experiments the cortex was stimulated and the response of the astrocyte Em to the K^+^ released from neighbouring neurons was recorded. The astrocytes responded in a qualitatively predictable manner, but quantitatively the changes were not as predicted by the Nernst equation. Elevations in interstitial K^+^ are not sustained and K^+^ returns to baseline rapidly due to the buffering capacity of astrocytes, a phenomenon studied by Bruce, and his son Chris, published 27 years after Bruce’s initial publications. Thus, a lifetime spent investigating K^+^ buffering has seen enormous advances in glial research, from the time cells were identified as ‘presumed’ glial cells or ‘silent cells’, to the present day, where glial cells are recognised as contributing to every important physiological brain function.

## Introduction

The opportunity to pay tribute to Bruce Ransom is one that I relish. I have known Bruce since 1996, when I started a post-doctoral position in his new labs at the University of Washington, Seattle, on his move from Yale to take up the Warren and Jermaine Magnuson Chair in Medicine for Neuroscience. I stayed at the University of Washington until 2002, when I returned to the UK, to my current job at the University of Nottingham. However, such biographical detail betrays none of the enjoyment and excitement of what it was like to work with Bruce; it is no exaggeration to say that the years I spent with Bruce rank among the most fun, productive, and satisfying of my 30 year scientific career. Bruce’s enthusiasm is infectious and his mentorship was light touch, always there if you needed advice, but no pressure to perform. He allowed you to develop in your own time, trusting in his judgment that (a) he had hired the right person, and (b) that your own drive and intellectual curiosity would propel the research forward.

Bruce entered Medical School in 1967 and graduated with an M.D. and Ph.D. in 1972 from Washington University in St. Louis. He was able to graduate within 5 years at a time when most combined degrees took 7–8 years. Bruce commenced experiments in the laboratory of his supervisor, the esteemed neurosurgeon Sidney Goldring. Bruce’s technically challenging experiments (intracellular recording from cat cortex in vivo) were completed in a frenzy of effort from the summer of 1969 until January 1971, the remainder of his time in the lab devoted to writing manuscripts and his thesis. His first days in the lab proved to be very frustrating, an initial obstacle being the elementary process of making up stock solutions. Bruce would pile all the chemical bottles onto a lab cart and trundle down the hall to one of the other PI’s labs, where he would painstakingly weigh out components to the amusement of more experienced scientists. Bruce worked in a small barren room with a dense shade over the single small window to keep out light, so he could see the oscilloscope and film the events with a ‘Grass Camera’ that ran across the screen. He occasionally slept on the desktop in the shared office space if he had a long evening preparing figures. A window air conditioner struggled to keep the temperature under 85 °F in the heat of a St. Louis summer. The greatest challenge Bruce faced was to pull intracellular electrodes, which he used to plunge into the beating cortex of anesthetized cats to record from cortical cells. Making the electrodes was ridiculously hard and capricious and it took a few months to master basic lab protocols and learn how to pull usable electrodes [1]. In the machine shop Bruce received instructions about the machines used for turning blocks of plastic into useful appliances, but was left to his own ingenuity to fashion the required objects for his experiments. He built a tiny chamber that had electrodes at the bottom, a crude perfusion system and a very smooth opening at the bottom, which was carefully placed on the cortex to dampen pulsations and create a small pool of fluid whose ionic composition he could control. It took most of a long morning to obtain an experimental animal, carry out the significant surgery needed to expose the cortex, dissect away the dura, and position his homemade chamber gently onto the cortical surface so that he could perfuse the small chamber with solutions of variable ionic concentrations. Each experiment started with maximum optimism and excitement - today would be the day! Finally in the early spring of 1970, Bruce started to impale cells reliably with recordings that could last an hour or more.

Bruce’s Ph.D. work was published sequentially as three papers in the September 1973 issue of the Journal of Neurophysiology [2–4]. I have chosen to reflect on the first papers that Bruce published, not just because these papers heralded the arrival of a new and vital talent on the scene, but because they set Bruce on a career path from which he has not deviated in more than 45 years, that of establishing functional roles for glial cells. These papers resonate as they evoke the sense of struggle, followed by conquest and finally achievement that all scientists experience throughout their careers. The rationale behind these papers was based on observations that Sidney Goldring had made in the 1960s, where he stimulated the cortical surface of the brain of an anesthetized cat and recorded slow negative potentials with extracellular electrodes [5]. In addition, Goldring had made intracellular recordings from cells that displayed relatively hyperpolarized membrane potentials and did not show spontaneous synaptic potentials or action potentials. These cells, so-called “silent cells” (presumably astrocytes), would depolarize during cortical stimulation with a duration that matched those of the extracellularly recorded slow, negative potentials. Goldring was interested in elucidating the mechanism(s) underlying these observations. Goldring was well aware of the pioneering studies of Stephen Kuffler and colleagues on the properties of glial cells in the optic nerve of the amphibian *Necturus* [6, 7], and he deduced that these studies were highly relevant to his own observations. (Enter Bruce Ransom!)

## Initial Experiments

**Fig. 1:**
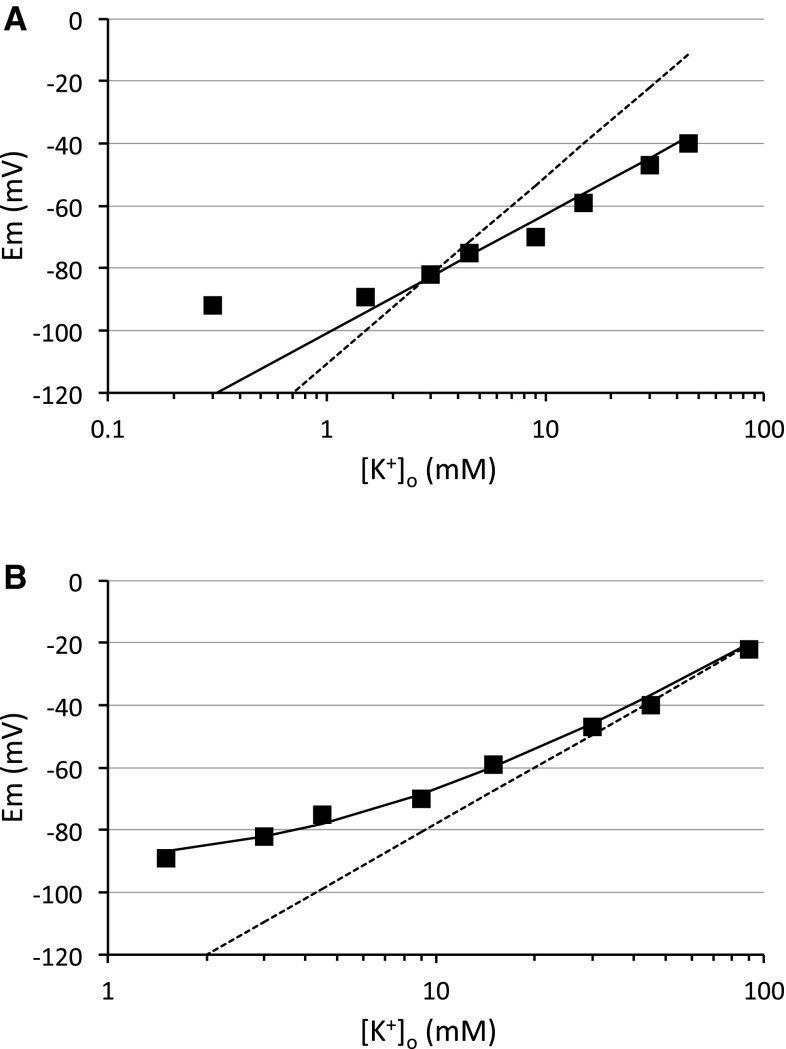
Relationship between [K^+^]_o_ and Em in in situ cat cortical astrocytes. **a** Reproduction of Fig. 2 from [4] showing experimentally acquired data (*filled square*), Nernstian relationship of the best fit of the data at concentrations of [K^+^]_o_ greater than 3 mM with a slope of 38 mV (*straight line*), and Nernstian relationship with a slope of 60 mV calculated such that the value at 3 mM [K^+^]_o_ matches the experimentally acquired data (*dotted line*). **b** Experimentally acquired data as in A (*filled square*), Nernstian relationship according to Em = 60 log ([K^+^]_o_/200) (*dotted line*), and GHK relationship drawn as ${Em }={ 6}0{ log }\left( {{{\left[ {{{K}^+}} \right]}_{o}}+0.0{38} \times {{\left[ {{N}{{a}^+}} \right]}_{o}}} \right)/{2}00)$ where [K^+^]_o_ + [Na^+^]_o_ = 150 mM (*curved line*)

Stephen Kuffler, one of Bruce’s scientific heroes, was a polymath with an insatiable quest for knowledge. In the mid 1960s he turned his attention to defining roles for glial cells, about which almost nothing was known. Kuffler realized that the electrophysiological techniques of the day were inadequate to record reliably from the mammalian preparations, thus he elected to use the optic nerve of the mudpuppy *Necturus* as a model system. This offered the advantages of an easily accessible tissue that was sufficiently robust to endure the extended periods of recording required by the experimental protocol. The optic nerve could be recorded from under both in vitro and in vivo conditions, providing a relevant and comparative physiological basis for the experiments [6]. One of Kuffler’s main findings, published in two sequential papers in the Journal of Neurophysiology in 1966 [6, 7], was that the membrane potential (Em) of presumed glial cells (i.e. cells with large, hyperpolarized membrane potentials) varied logarithmically with changes in extracellular K^+^ concentration in a manner predicted by the Nernst equation, indicating that glia are exclusively permeable to K^+^. Stated another way, the Em of glia in *Necturus* optic nerve is determined by the equilibrium potential for potassium ions (E_K_). The experiments described in Bruce’s first paper [4] were aimed at determining whether the mammalian glial cells possessed similar characteristics. The recording technique that Bruce faced was considerably more complex than Kuffler’s, and he initially struggled for a few months to make reliable recordings. In this venture he was helped by Carl Rovainen, a co-worker of Kuffler, who helped him to fashion glass micro-electrodes suitable for recording from small mammalian glial cells. A craniectomy was performed on the skull of an anesthetized cat and the underlying brain exposed. An agar molded circular barrier was placed on top on the brain to form an enclosed reservoir into which the aCSF was introduced (Fig. 1 of [4]). Sharp electrodes were inserted into the cortical surface to depths of up to 400 µm, although 70% of the recordings were from cells in the upper 200 µm. In the initial experiments, due to the difficulty in recording from cells for durations sufficient to allow exchange of aCSF containing multiple concentrations of K^+^, Bruce applied to the cortical surface aCSF containing the desired [K^+^] prior to penetrating the cell, thus recording Em at only one [K^+^]. Such a protocol allowed Bruce, over time, to accumulate data from many cells at a variety of [K^+^] (0.3, 1.5, 3, 4.5, 9, 15, 30, 45 mM), with between 9 and 34 cells recorded from at each concentration (Fig. 2 of [4]). The results were not as expected in that although a linear response in Em was recorded relative to [K^+^] plotted on a logarithmic scale, the slope was not as steep as that predicted by the Nernst equation, on average 38 mV compared to the Nernstian slope of 60 mV (Fig. 1a). However the experience gained in carrying out these initial experiments enabled Bruce to subsequently record from cells for extended durations, allowing multiple solution exchanges of aCSF containing [K^+^] between 1.5 and 45 mM during a single recording. These data continued to show a slope of between 24 and 43 mV, again lower than expected (Fig. 5 of [4]). One of the technical issues that clouded interpretation of the data was the fact that based on the Nernst equation, where Em = 0 mV, [K^+^]_o_ = [K^+^]_i_. Under these circumstances [K^+^]_i_ was calculated (assuming a purely K^+^-permeable membrane) as 420 mM, an unreasonably large estimate. Given the instability of Em that accompanied high [K^+^]_o_ no accurate estimates of [K^+^]_i_ were available, and thus the predicated Nernstian relationship was elusive. Such results must have been disappointing given the difficulty required in acquiring the data, but probable explanations for the results (see below) offered important insights into several key properties of astrocytes that would subsequently be revealed over the next decade.

## When Em ≠ E_K_

One explanation for the disparity between the experimentally recorded Em and the predicted value (E_K_) is that astrocyte membranes are permeable to additional ions other than K^+^. Bruce investigated this and found no effect of changing Ca^2+^ on Em, and the Em responded in a quantitatively similar manner to addition of KCl or K_2_SO_4_, implying that the membrane was impermeable to both of these anions [4]. The putative permeability to Na^+^ was more complex to investigate. It was estimated that the glial [Na^+^]_i_ was about 25 mM [8] with [Na^+^]_o_ about 150 mM in situ. Na^+^ was substituted with equimolar choline. The low number of cells recorded from (4), and the variability of the effects on Em suggested little permeability to Na^+^, but a finite permeability to Na^+^ could not be excluded. The relationship describing the equilibrium potential for a membrane permeable to K^+^ and other ions was initially derived by Hodgkin and Katz in their classic 1949 paper [9], and is modelled by the Goldman Hodgkin Katz (GHK) voltage equation. This equation is simply an extension of the Nernst equation that adds the concentration ratio of additional permeant ions scaled by their permeability relative to K^+^ [9]. In Fig. 6, the relationship between [K^+^]_o_ and Em was plotted showing a deviation from the predicted Nernstian relationship of Em = 60 log ([K^+^]_o_/200). The data were best fit by a GHK equation with a permeability ratio of K^+^:Na^+^ of 1:0.038 (redrawn as Fig. 1b). The GHK equation was expressed as Em = RT/F ln (([K^+^]_o_*p[Na^+^]_o_)/[K^+^]_i_); Bruce and Sidney no doubt expected that anyone capable of appreciating their work would realize that the product of p and [Na^+^]_i_ could be omitted on account of its negligible value.

## The Astrocytic Syncitium

**Fig. 2:**
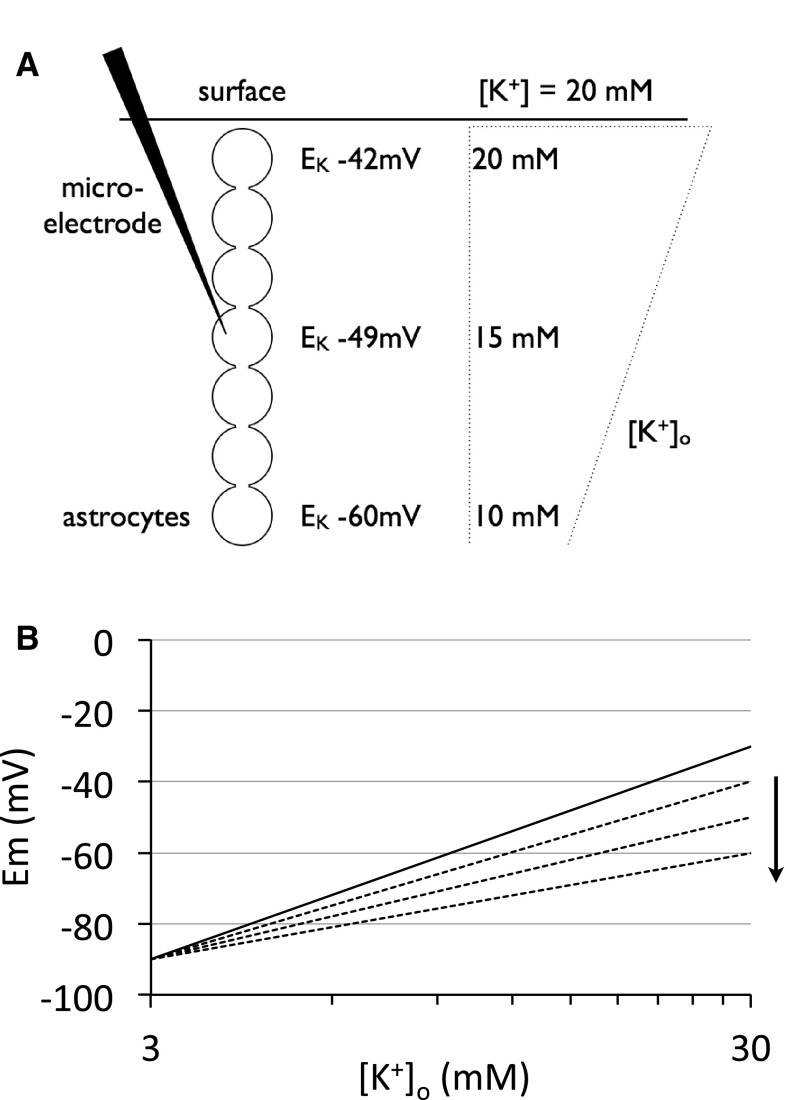
Schematic illustrating the effects of the diffusion barrier created by the interstitial space and a functional astrocyte syncitium on [K^+^]_o_ deep in the cortex. **a** The right hand side of the figure demonstrates the effects of the diffusion barrier created by the tortuosity of the interstitial environment in combination with the powerful astrocytic buffering capacity that leads to a mis-match between applied [K^+^] in the aCSF bathing the cortical surface and the actual [K^+^]_o_ in the interstitial fluid deep into the cortex (enclosed in *dotted box*). The calculated E_K_ at various depths based on estimated [K^+^]_o_ (*right side*) are shown. The astrocyte syncitium (*left*) illustrates multiple inter-connected astrocytes. **b** Hypothetical relationship between Em and [K^+^]_o_ in astrocytes recorded at various depths in the cat cortex. The *straight line* depicts the Nernst relationship of Em = 60 log ([K^+^]_o_/[K^+^]_i_). The *dotted lines* depict the Em of astrocytes recorded at increasing depths from the surface, the shallowest slope corresponding to the greatest depth

In addition to issues of membrane permeability there were two separate factors, which could conspire to result in Em at variance from that predicted by the Nernst equation. The first is that the value of [K^+^] in the aCSF bathing the cortical surface may not match the value of interstitial [K^+^]_o_ in the vicinity of the recording, due to the diffusion barrier presented by the brain parenchyma. This situation would be exacerbated for cells recorded at greater depths below cortical surface because of the formidable diffusion barrier presented by the tortuosity of extracellular space [10]. Einstein’s law of diffusion states that the time taken for a molecule to diffuse a certain distance is proportional to the distance squared [11]. Given the limited duration of exposure of the brain to each [K^+^] in the aCSF, coupled with the powerful buffering capacity of astrocytes (see later), it is unrealistic to expect that at distances of up to 400 µm from the cortical surface adequate penetration of K^+^ would occur such that the aCSF and interstitial [K^+^]_o_ would be in equilibrium [10]. Since the interstitial in situ [K^+^]_o_ is about 3 mM, the higher the concentration of aCSF [K^+^], the greater the attenuation of the actual interstitial [K^+^]_o_, a phenomenon that would result in more hyperpolarized values of Em being recorded, resulting in a relationship between Em and [K^+^]_o_ with a slope lower than predicted (Fig. 2a). The second factor is the presence of gap junctions that connect multiple astrocytes into a syncitium [12], the low resistance pathways between astrocytes conferring free passage of voltage, ions, and small molecules between astrocytes [13]. In this situation, the Em recorded from a single astrocyte is actually the aggregate Em of multiple astrocytes. Although in 1971 such concepts were difficult to test, they have since been established and offer an additional explanation as to the deviation of Em from E_K_. Imagine that numerous astrocytes are interconnected in a syncitium. In response to modest increases in aCSF [K^+^] the cells deeper in the cortex would express a more hyperpolarized Em than more superficial cells, but given the voltage sharing between cells, the deeper cells would ‘pull’ the Em recorded to more hyperpolarized values (Fig. 2b). The relationship between variations in localized interstitial [K^+^]_o_ and interconnected astrocytes was the basis for the concept of spatial buffering of K^+^ proposed by Richard Orkand in the 1980s and first suggested by Kuffler in 1966 [14, 15]. This concept uses the mismatch between Em and E_K_ to predict influx of interstitial K^+^ into astrocytes at regions of high localized interstitial [K^+^]_o_, where E_K_ is more depolarized than the astrocytic Em. At more distant regions of the syncitium where interstitial [K^+^]_o_ is low, E_K_ is more hyperpolarized than Em, thus promoting K^+^ efflux from astrocytes. This process thus distributes K^+^ away from regions of high interstitial concentration.

## Stimulus and Em

The experiments described in Bruce’s second paper [3] further examined the relationship between [K^+^]_o_ and Em, this time in response to electrical stimulation. Kuffler had carried out similar pioneering studies in the 1960s, where he attempted to address the Em depolarizations that occur in glial cells in response to stimulation of *Necturus* optic nerve [7]. The reasoning behind such experiments is as follows. The Hodgkin and Huxley model of the propagated action potential [16] predicted efflux of K^+^ from axons during the repolarizing phase, which they confirmed by measuring radioactive flux of K^+^ across axon membranes [17]. The advent of ion-sensitive microelectrodes with tips sufficiently small to record K^+^ in the interstitial space [18] confirmed and quantified the degree of interstitial K^+^ accumulation that results from axonal firing [19]. Although calculations predict that the fluxes of Na^+^ and K^+^ that produce action potentials are in the fractions of a millimole [20], given the relatively small interstitial volume, the non-inactivating nature of the delayed rectifier K^+^ channels, and the low resting value of K^+^ (3 mM) substantial interstitial K^+^ accumulates as a result of trains of action potentials evoked in axons [19]. The restoration of K^+^ levels will be covered later, but it is germane to point out that as a general principal, the higher the frequency of stimulus the greater both the magnitude and rate of [K^+^]_o_ increase. Given the sensitivity of *Necturus* glial cells to extracellular K^+^ [6], the Em response to stimulation allows the size of stimulus-induced elevations in [K^+^]_o_ to be estimated. Stimulus of the optic nerve with sequential identical stimuli would be predicted to release identical amounts of K^+^ from the axons, to which the glial cell membrane should respond with a depolarization that can be measured with microelectrodes. At the heart of these responses is the logarithmic function, and the fact that a K^+^-selective membrane should depolarize in a predictable manner to changes in [K^+^]_o_. Kuffler and colleagues used the relationship that Em = E_K_ = 59 log ([K^+^]_o_/[K^+^]_i_) to extrapolate measurements of Em to [K^+^]_o_ (assuming a fixed value of [K^+^]_i_ there are only two variables, Em and [K^+^]_o_). Kuffler was able to extrapolate that an Em depolarization of 2.1 mV resulted from a [K^+^]_o_ increase of 0.25 mM produced by the first stimulus, and was thus able to predict the attenuating stepwise depolarizations in Em resulting from subsequent stimuli, each of which liberated the same amount of K^+^ (Fig. 6 of [7]).

**Fig. 3:**
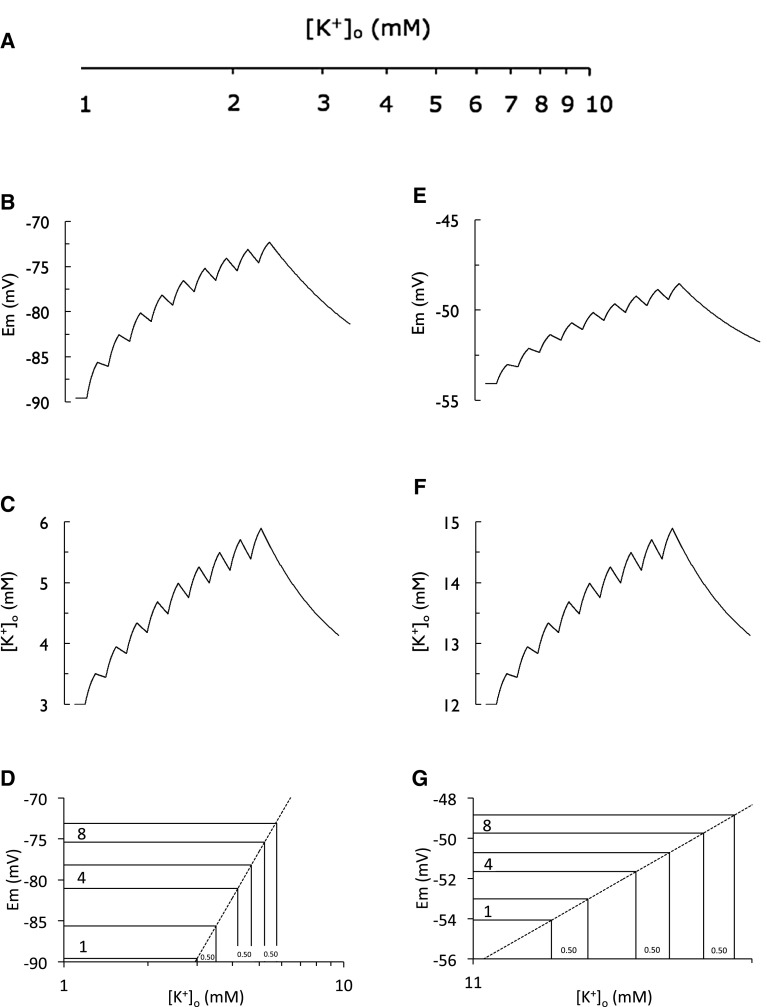
Attenuation of the Em depolarization resulting from sequential stimuli in various bath aCSF [K^+^]. **a** Concentrations of [K^+^] from 1 to 10 mM plotted on a logarithmic scale. **b** A model based on Em = 60 log ([K^+^]_o_/99), illustrating the calculated Em depolarization of the astrocyte Em in response to nine sequential stimuli at 8 Hz, where each stimulus liberates the same amount of K^+^. **c** The elevation on [K^+^]_o_ that produce the Em depolarization in **b**. Note how each stimulus raises [K^+^]_o_ by the same amount. **d** Calculated amount of K^+^ released per stimulus for the 1st, 4th and 8th stimuli, plotted relative to the corresponding Em depolarization. **e**–**g**, as in **b**–**c** with the exception that the baseline [K^+^]_o_ was modelled as 12 mM

We have already discussed Bruce’s finding that Em of ‘silent cells’ in cat cortex deviated from the Nernstian prediction, so rather than reproduce Bruce’s data, I will instead deploy a model of Em for illustrative purposes and clarity. The model is based on a [K^+^]_i_ value of 99 mM, RT/F = 60 mV, and we assume that each stimulus releases an identical amount of K^+^, which causes a predictable depolarization of Em based on the Nernst equation. On cessation of the stimulus the Em falls back towards baseline with an exponential time course due to buffering of K^+^ (see later). A train of stimuli results in a characteristic serrated profile of Em depolarization [7]. Although E_K_ is a concept, it can be assigned a value and if we assume that astrocytes are selectively permeable to K^+^, then E_K_ = Em. Because the value of Em can be recorded with microelectrodes we can calculate the value of [K^+^]_o_, as Kuffler did. Thus, the Nernst equation simplifies to two variables, [K^+^]_o_ and Em, and if we know one we can calculate the other. In this particular case we model the elevation in [K^+^]_o_ and calculate the resulting Em. In the experiment illustrated we have modelled the [K^+^]_o_ in the CSF as 3 mM, [K^+^]_i_ as 99 mM, thus Em = −89 mV. A train of 9 stimuli imposed at a rate of 8 Hz was imposed. Our model shows a serrated profile of Em in response to the successive stimuli (Fig. 3b). The Em depolarization resulting from the stimulus-evoked elevations in [K^+^]_o_ (Fig. 3c) attenuate for each successive stimulus, such that the Em evoked by the 8th stimulus is considerably less than that evoked by the 1st (Fig. 3b). The increases in [K^+^]_o_ that produce the depolarizations are illustrated in Fig. 3c, and show that each stimulus causes [K^+^]_o_ to increase by the same amount, in this case 0.5 mM. In order to explain the attenuation of successive depolarizations we must appreciate that based on the Nernst equation, Em = E_K_ = 60 log ([K^+^]_o_/[K^+^]_i_). However in these calculations 60 and [K^+^]_i_ are constants, thus the relationship simplifies to Em ~ log [K^+^]_o_. We can now directly visualize the relationship between Em and [K^+^]_o_ by plotting [K^+^]_o_ between 1 and 10 mM on a log scale (Fig. 3a). Given that each successive stimulus releases the same amount of K^+^, [K^+^]_o_ will increase by a fixed amount for each stimulus, thus it is relatively straightforward to visualize the attenuation in Em depolarization resulting from successive stimuli. Stated another way, if baseline [K^+^]_o_ is 1 and each stimulus caused [K^+^]_o_ to increase by 1 mM then Em, which is related to log [K^+^]_o_, would clearly attenuate for successive stimuli, as indicated by the decremental decrease in the progression of stepwise increases in [K^+^]_o_ of 1 mM along the logarithmic scale (Fig. 3a). The data (Fig. 3b) shows the experimentally-modelled consequence of this relationship, and can be clarified by using the assumption that Em = E_K_ to calculate how much K^+^ is released during each stimulus as (−80−3.8) = 60 log (([K^+^]_o_−3)/99). For a more accurate correlation between the stimulus induced increase in Em and [K^+^]_o_, a plot of the Em depolarization prior to the decrease towards baseline for each of the stimuli provides clarification as illustrated in Bruce’s original paper (Fig. 6 of [3]). We have not included all nine stimuli but only the 1st, 4th and 8th to illustrate the point. Measuring the depolarization of Em caused by each stimulus and plotting them against the appropriate part of the Nernstian relationship allows us to read off the corresponding increase in [K^+^]_o_ that produced the Em depolarization, in each case 0.5 mM K^+^ (Fig. 3d).

As a further test of the relationship between Em and [K^+^]_o_, Bruce and Sidney predicted that if the baseline aCSF [K^+^] was increased then the resulting Em depolarizations in response to each stimulus would be smaller than those that occur when aCSF [K^+^] was 3 mM. These experiments proved difficult to carry out due to the difficulty in changing aCSF whilst reliably maintaining stable intracellular recordings. However the two cells that were successfully recorded from showed that when 3 mM [K^+^] aCSF was replaced with 15 mM [K^+^] aCSF the magnitude of the Em depolarization was decreased from 14 to 7.5 mV (Fig. 7 of [3]). Again the results were qualitatively as expected but given the smaller than predicted changes in Em in response to changes in known K^+^ quantitative, definitive conclusions could not be made. I have modelled this in Fig. 3e–g to show the quantitatively similar elevations in [K^+^]_o_ in response to individual stimulus in aCSF with [K^+^] of 3 or 12 mM (Fig. 3c, f). However the baseline Em depolarizes from −89 to −54 mV in the presence of 12 mM [K^+^] and the Em depolarizations are attenuated compared to those predicted in 3 mM K aCSF (Fig. 3e).

## Buffering of Interstitial K^+^

These experiments highlighted the elevations in [K^+^]_o_ that are produced by neuronal firing in the cat cortex, and the resulting effects on the Em of astrocytes whose membranes are permeable to K^+^ [3]. Because increased [K^+^]_o_ depolarizes neurons and can have both excitatory and inhibitory effects [20], via Na^+^ channel inactivation and other mechanisms [20], normal brain function requires tight regulation of activity-dependent changes in [K^+^]_o_. The remainder of this appreciation will be devoted to mechanisms that return [K^+^]_o_ to normal levels and the fate of K^+^ once released from axons, the rate of restoration providing an index of buffering capacity. The effects of these buffering mechanisms shape the slow transient depolarizations and subsequent rapid repolarization towards resting Em during and after electrical stimulation (Fig. 2 of [3]). This may be compared to increasing the aCSF [K^+^] bathing the tissue, where the continuous presence of K^+^ overwhelms the buffering capacity and the Em depolarization is maintained (Fig. 4 of [4]).

**Fig. 4:**
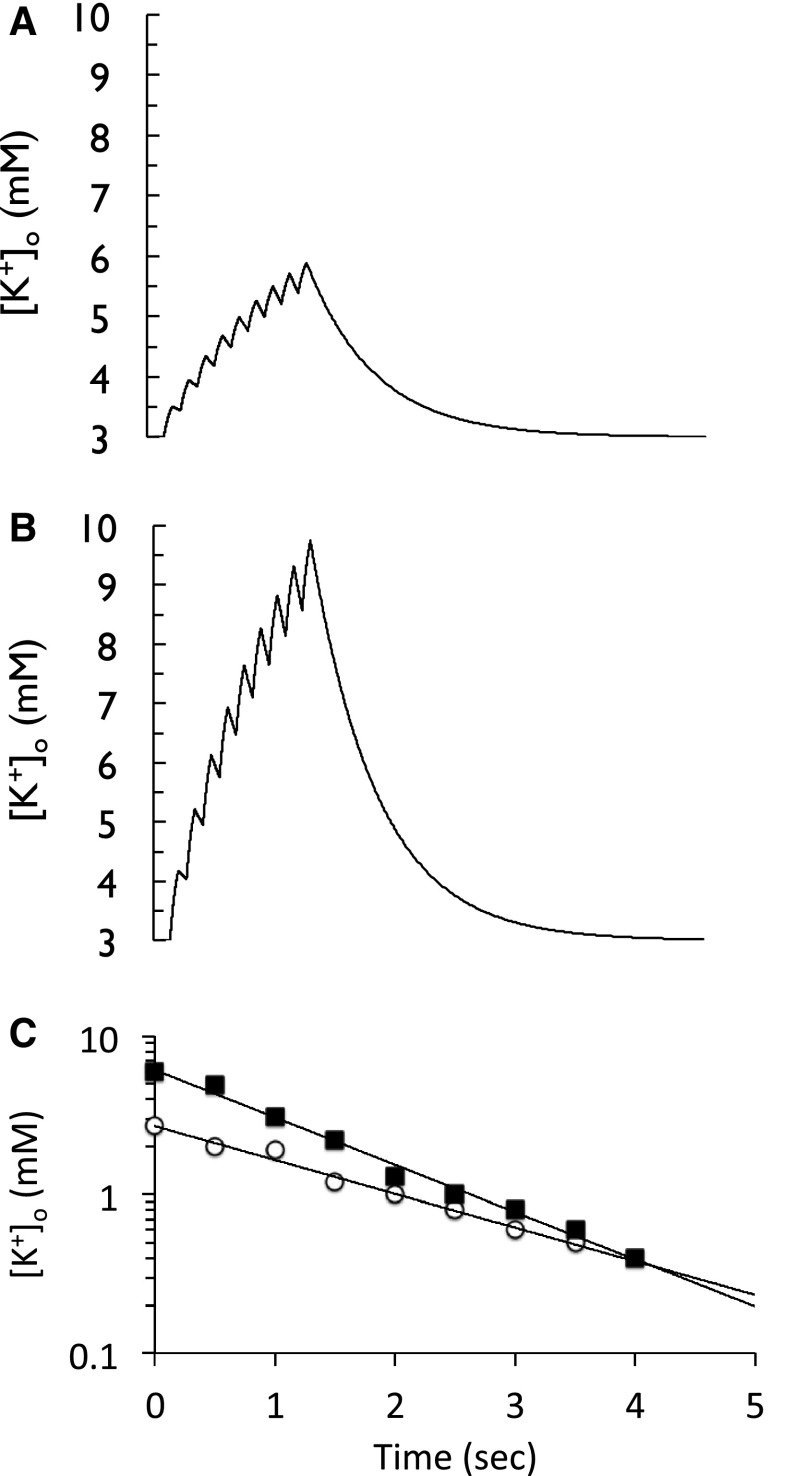
Return of interstitial [K^+^]_o_ towards baseline following a train of 9 stimuli. **a** and **b** The elevations in [K^+^]_o_ that result from two stimuli, and cause stepwise elevations in [K^+^]_o_ of 0.50 and 1.17 mM, respectively. On cessation of the stimuli the [K^+^]_o_ returns to baseline following a single exponential time course, which can be linearised by plotting [K^+^]_o_ on a logarithmic scale. **c** Note how the rate of [K^+^]_o_ clearance is greater for the larger increase in [K^+^]_o_, suggesting an activity dependent system is present

The effect of buffering on [K^+^]_o_ was quantified by converting Em to [K^+^]_o_ and plotting the result on a logarithmic scale versus time (Fig. 8 of [3]). The response of [K^+^]_o_ to two stimuli are shown, with one displaying a greater increase in [K^+^]_o_ to stimulus than the other, Fig. 4a, b, respectively. The rate of decrease of [K^+^]_o_ towards baseline also varied, the larger increase in [K^+^]_o_ displaying faster return to baseline. I have chosen to model this effect, as described above, and the results are shown in Fig. 4. The effects of optic nerve stimulus are shown in Fig. 4a, b, where the increases in [K^+^]_o_ are small (Fig. 4a) or large (Fig. 4b). Plotting the [K^+^]_o_ shows the relative increases in [K^+^]_o_ evoked by the individual stimuli as previously shown in Fig. 3. On cessation of stimulus the [K^+^]_o_ falls towards baseline with an exponential time course, the slope of the larger response greater than that of the smaller response (Fig. 4c). When these experiments were carried out in the early 1970s there was no established model for buffering, besides a general agreement that neurons must reclaim the K^+^ lost during stimulation. The rapid linear decrease in calculated [K^+^]_o_ is evidence of a powerful buffering system that restores ion homeostasis. Some of the interstitial accumulation of K^+^ may leak away but active processes are clearly involved [3]. I have chosen not to comment in any detail on the results contained in Bruce’s third paper [2], which concerns the slow hyperpolarization that occurs in the aftermath of high frequency direct cortical stimulation and follows the slow depolarization described above, since aspects of this observation are pertinent to the discussion below.

## A Family Affair

This issue of K^+^ buffering was addressed in detail in a paper [21], in which Bruce collaborated with his son Chris, born in 1970 during the heady days of Bruce’s initial triumphs in the lab, who has followed in his father’s footsteps to become a Neurologist. In these studies carried out at the University of Alabama, Chris was supervised by Dr Harald Sontheimer, formerly a post-doc in Bruce’s lab. This paper was published 27 years after Bruce’s initial papers, where the inexorable progress of technological advances had led to the development of techniques that allowed for more accurate recordings of [K^+^]_o_, namely K^+^ sensitive micro-electrodes. These micro-electrodes are fashioned from capillary glass tubing and possess at the tip a K^+^ sensitive resin [18]. The tip diameter is beveled to about 2 µm, whose hypodermic needle type profile permits insertion into the rat optic nerve with minimal disruption to the tissue, and measurement of interstitial K^+^ with a rapid response time [21]. The in vitro rat optic nerve offered a more controlled model than the in vivo cat cortex, with robust recordings of extended durations possible [22]. The study aimed at investigating the mechanism(s) responsible for the restoration of interstitial [K^+^]_o_ towards baseline following axonal stimulus.

**Fig. 5:**
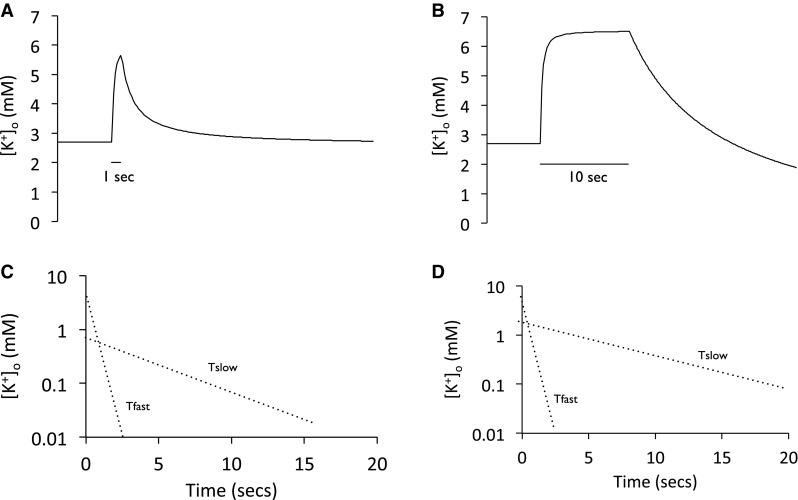
Recovery of [K^+^]_o_ towards baseline following stimulus in the rat optic nerve. **a** A short duration stimulus (1 s) induces a transient [K^+^]_o_ elevation that rapidly decreases on cessation of stimulus. **c** Plotting [K^+^]_o_ on a logarithmic scale can resolve the decay of [K^+^]_o_ into two phases, quantified as T_fast_ and T_slow_, with values of 0.48 and 4.0 s, respectively. **b** A longer duration stimulus of 10 s allows [K^+^]_o_ to plateau. **d** The decay of [K^+^]_o_ resolves into two phases, with time constants of 0.55 and 7.8 s

**Fig. 6:**
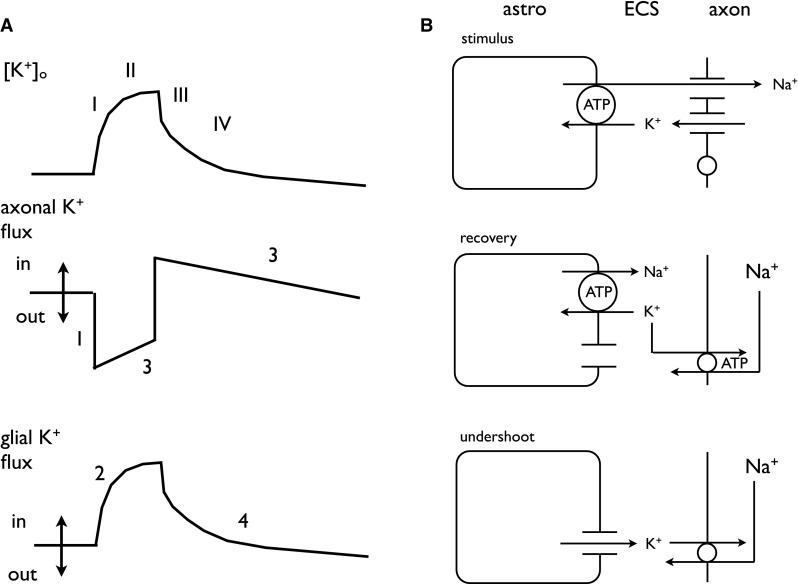
A model of [K^+^]_o_ buffering in rat optic nerve. **a** Relationship between [K^+^]_o_ and axonal and glial Na^+^ pump activity. The changes in [K^+^]_o_ during and after cessation of stimulus can be resolved into 4 phases; (I) the increase at the onset of stimulus, (II) the plateau of [K^+^]_o_, (III) the rapid decrease in [K^+^]_o_ followed by a slower (IV) decrease (*upper panel*). At the onset of stimulus K^+^ leaves the axon via voltage gated K^+^ channels, but this slows as intra-axonal Na^+^ accumulates. On cessation of stimulus the flux of K^+^ changes in response to activation of the axonal Na^+^ pump. There then follows an attenuating uptake of K^+^ into axons (*middle panel*). The glia Na^+^ pump is activated very rapidly following onset of stimulus, but the rate of influx decreases on cessation of stimulus, such the eventually K^+^ leaves the glia in response to the hyperpolarization caused by axonal uptake of K^+^ such that K^+^ decreases below baseline levels (*lower panel*). **b** K^+^ efflux from axons during stimulus leads to elevations in [K^+^]_o_ that activate the glial Na^+^ pump (*upper panel*). During recovery the axons take up Na^+^, which leads to activation of the axonal Na^+^ pump (*middle panel*). During the undershoot astrocytes leak K^+^ into the ECS that keeps the axonal Na^+^ pump activated until [K^+^]_o_ returns to baseline levels (*lower panel*)

**Fig. 7:**
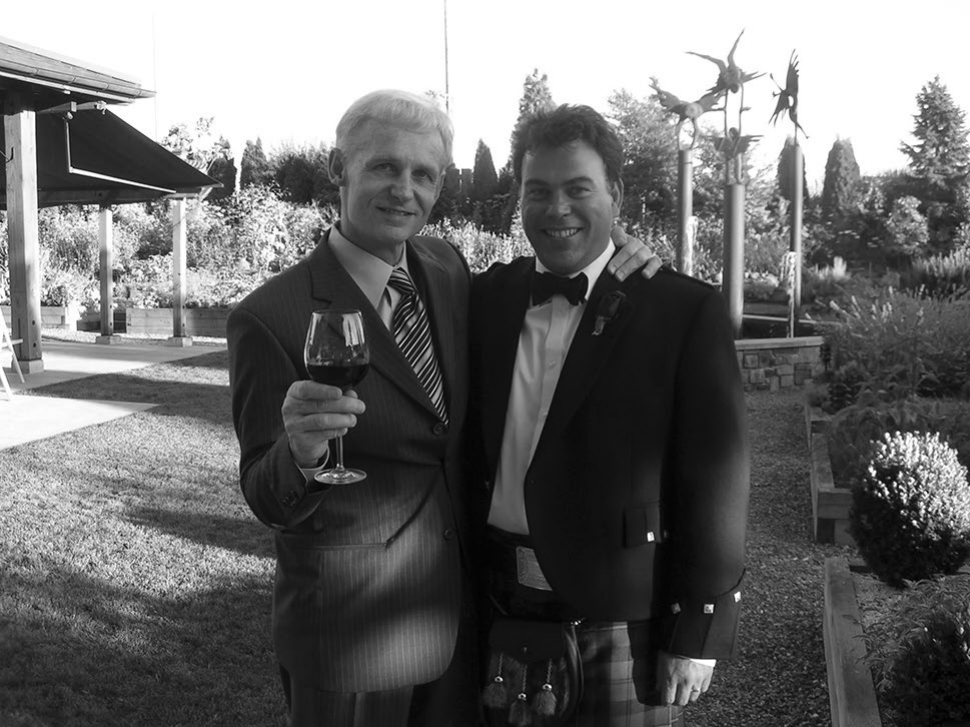
In addition to the academic benefits of my association with Bruce, I met my wife at the Department of Neurology in Seattle, where she worked as a student helper assisting Catherine Dinnie (another Scot) in the Neurology Residency program. Dr Bruce Ransom (glass of wine in hand) at the author’s wedding, Willows Lodge, Seattle, July 30th 2005

The decrease in [K^+^]_o_ following short (1 s) or prolonged stimuli (10 s) was quantified via curve fitting techniques (Fig. 2 of [21]). The decay in [K^+^]_o_ was assessed to occur in two phases since it was best fit by 2 exponentials, named T_fast_ and T_slow_, reflecting their relative half times. The resolution of the decay into two phases was most obvious following the 1 s stimulus. The first phase rapidly lowered [K^+^]_o_ at a rate of 0.78 mM sec^−1^, the second and slower phase restoring [K^+^]_o_ at rate of 0.061 mM sec^−1^. The first phase was inhibited by Na^+^ pump blockers, and given the sensitivity of the astrocytic Em to increases in [K^+^]_o_, is likely due to the astrocytic Na^+^ pump taking up K^+^. The second slower phase was due to the axonal Na^+^ pump, a slower response initiated by the accumulation of Na^+^ in axons during action potential firing, and this component is also sensitive to Na^+^ pump block (Fig. 5). In response to small durations of stimuli the majority of the [K^+^]_o_ restoration occurs via the glial Na^+^ pump, represented by the fast time constant, since short duration stimulus would not increase intra-axonal Na^+^ sufficiently to activate the axonal Na^+^ pump. However, increased durations of stimulus activate the axonal Na^+^ pump, hence the larger relative contribution of T_slow_ to K^+^ clearance, since more [Na^+^]_i_ accumulates in axons and it is then pumped out of the axon. Activation of this axonal Na^+^ pump led to an undershoot in [K^+^]_o_ below baseline, the result of strong activation of the axonal Na^+^ pump resulting in uptake of K^+^ into the axons. This was subsequently counteracted by release of K^+^ from astrocytes via K^+^ channels (Fig. 6). Such an undershoot of [K^+^]_o_ (seen as an afterhyperpolarization of Em) was described in the third of Bruce’s initial papers [2], where it was reported as dependent upon the duration of stimulus and considered due to activation of the axonal Na^+^ pump. Note that in this early paper there were no recordings of [K^+^]_o_, which could only be estimated from measurements of Em via the logarithmic relationship between Em and [K^+^]_o_ (Fig. 7).

## Role of Interstitial K^+^ as Regulator of Neuronal-Glial Metabolic Interactions

When I started to work with Bruce in July 1996, our focus was on measuring the perturbations in interstitial Ca^2+^ associated with anoxia in rat optic nerve, using Ca^2+^ sensitive micro-electrodes [23]. However our experimental focus changed tack in 1997 upon publication of studies carried out by Bob Fern, at the time an Assistant Professor, who had accompanied Bruce from Yale. Bob and Bruce found that in response to aglycemic conditions, the compound action potential (CAP) recorded from rat optic nerve was sustained for 30 min before it failed [24], compared with the 5 min latency to CAP failure that occurs in response to anoxia [25]. These observations opened up highly productive research into the role of brain glycogen. Bruce and I have published extensively in this area, but this is not the appropriate arena to review this research. However, there is a very pleasing symmetry in that recent studies have implicated a role for interstitial K^+^ in regulating the metabolic interactions between neurons/axons and astrocytes. Our work had clearly shown that astrocytic glycogen was broken down to lactate [26], which was subsequently trafficked to axons [27] during periods of a/hypoglycemia and increased tissue energy demand. The missing part of the puzzle was identifying the signaling mechanism by which astrocytes sensed increased neuronal activity. We had initially proposed that elevations in [K^+^]_o_ would be an ideal signal to coordinate astrocytic metabolism to neuronal activity, and indeed carried out experiments with K^+^ sensitive micro-electrodes to detect aglycemic elevations in [K^+^]_o_ that could precipitate glycogen metabolism in astrocytes, but an obvious correlation was elusive [28]. Brian MacVicar, a close friend of Bruce, published a paper in 2012 that convincingly showed that physiologically-relevant elevations in interstitial [K^+^]_o_ promoted astrocytic glycogenolysis and subsequent transfer of lactate to neurons [29]. Such a mechanism has universal appeal since all neurons/axons in both the central and peripheral nervous systems show activity-induced K^+^ release. The K^+^ theory was reinforced by data acquired using FRET technology in an in vivo model showing that physiologically relevant increases in interstitial K^+^ promotes glycogenolysis and channel-mediated release of lactate, distinct from the monocarboxylate transporter [30].

## The Scottish Connection

In conclusion, and personal friendship not withstanding, over the course of the last 45 years Bruce has made an enormous contribution to the field of extracellular K^+^ regulation, in which time he has published 27 papers and 5 book chapters on the topic (see References section). To fully appreciate Bruce’s contribution to the field we need only refer to the title of his first published paper in 1973 “Ionic determinants of membrane potential of cells presumed to be glia in cerebral cortex of cat”, to realize that glial cells were described by what they were not (i.e. neurons), and classified by what they could not do (fire action potentials, hence the term “silent cells”). Entering such an unexplored and neglected area of research could be viewed as appealing as any data acquired would be worthy of interest, but this was counteracted by the prejudices of a neurocentric and skeptical world [31]. In no small part due to Bruce’s prescient and tireless championing of all things glia, neuroscience has now moved out of what Bruce has described as “the dark period when the neuron concept dominated brain science,” and there are now well-recognized and important roles for glia in literally every physiological and pathophysiological process in the brain.

Indeed it is a fair question to ask of the first researcher to penetrate an astrocyte with a micro-electrode “Were you more disappointed that it did not fire an action potential, than excited by its hyperpolarized membrane potential?” The application of the Nernst equation to describe the potential difference that results from ion gradients separated by a membrane containing selectively permeable ion channels was one of the most profound and far reaching in the short history of neuroscience, at the heart of which lies the logarithmic function. I have recently covered this topic in detail elsewhere [32] but national pride compels me to proclaim that the inventor of the logarithm was a Scotsman, John Napier, 8th Laird of Merchiston. The logarithm, proposed in 1614, during the lifetime of Shakespeare, was the foundation underlying mathematics for the next 400 years, and it is only the most recent generation of students who do not have to consult log tables to facilitate complex arithmetical calculations. I wonder how many of the researchers who will attend the XIII European Meeting on Glia Cells Conference in Edinburgh in July 2017, which Bruce will attend, will appreciate that in a castle, where Napier was born and died, in the Merchiston campus of Edinburgh’s Napier University, lies the foundation of an invention that played a critical role in ascribing the first physiological role to astrocytes, namely K^+^ buffering.
